# Spermidine improves angiogenic capacity of senescent endothelial cells, and enhances ischemia-induced neovascularization in aged mice

**DOI:** 10.1038/s41598-023-35447-3

**Published:** 2023-05-23

**Authors:** Daisuke Ueno, Koji Ikeda, Ekura Yamazaki, Akiko Katayama, Ryota Urata, Satoaki Matoba

**Affiliations:** 1 Department of Cardiology, Kyoto Prefectural University of Medicine, 465 Kajii, Kawaramachi-Hirokoji, Kamigyo, Kyoto, 602-8566 Japan; 2 Department of Epidemiology for Longevity and Regional Health, Kyoto Prefectural University of Medicine, 465 Kajii, Kawaramachi-Hirokoji, Kamigyo, Kyoto, 602-8566 Japan

**Keywords:** Experimental models of disease, Translational research

## Abstract

Aging is closely associated with the increased morbidity and mortality of ischemic cardiovascular disease, at least partially through impaired angiogenic capacity. Endothelial cells (ECs) play a crucial role in angiogenesis, and their angiogenic capacity declines during aging. Spermidine is a naturally occurring polyamine, and its dietary supplementation has exhibited distinct anti-aging and healthy lifespan-extending effects in various species such as yeast, worms, flies, and mice. Here, we explore the effects of spermidine supplementation on the age-related decline in angiogenesis both in vitro and in vivo. Intracellular polyamine contents were reduced in replicative senescent ECs, which were subsequently recovered by spermidine supplementation. Our findings reveal that spermidine supplementation improved the declined angiogenic capacity of senescent ECs, including migration and tube-formation, without affecting the senescence phenotypes. Mechanistically, spermidine enhanced both autophagy and mitophagy, and improved mitochondrial quality in senescent ECs. Ischemia-induced neovascularization was assessed using the hind-limb ischemia model in mice. Limb blood flow recovery and neovascularization in the ischemic muscle were considerably impaired in aged mice compared to young ones. Of note, dietary spermidine significantly enhanced ischemia-induced angiogenesis, and improved the blood flow recovery in the ischemic limb, especially in aged mice. Our results reveal novel proangiogenic functions of spermidine, suggesting its therapeutic potential against ischemic disease.

## Introduction

Population aging is a global health issue because of its close association with increased morbidity and mortality of various diseases such as cognitive and cardiometabolic disease. Anti-aging interventions aiming to extend healthy lifespan in mammals have been extensively investigated, and several lifestyle and pharmacological interventions have shown potent anti-aging effects in mammals. Although caloric restriction and exercise are widely considered the most popular anti-aging lifestyle interventions, it is often very difficult to sustain these lifestyle strategies^1^. Pharmacological interventions might be less problematic in this regard. Spermidine is a naturally occurring polyamine, the levels of which decrease with age in many organisms, including humans^1,2^. However, healthy nonagenarians and centenarians are notable exceptions to this decline in spermidine levels as their whole-blood spermidine concentrations are similar to those in younger (middle-aged) individuals^3,4^. These findings strongly suggest the crucial role of spermidine in aging and/or age-related diseases.

Spermidine supplementation has been found to extend the lifespan of yeast, flies, worms, and mice^3,5^. Furthermore, dietary spermidine improved cognitive function, and showed cardioprotective effects in aged mice^6,7^. It should be mentioned that enhanced longevity caused by dietary spermidine was critically associated with enhanced autophagy, because autophagy inhibition was found to cancel the various beneficial effects of spermidine^5,6^. Autophagy is a process that catabolizes intracellular components to maintain energy homeostasis and protect cells against stress, and many organisms show signs of decreased autophagic capacity with age^8^.

Cardiovascular disease is one of the major causes of death in many countries. Impaired angiogenesis caused by the aging process is causally involved in the high morbidity and mortality rates of ischemic cardiovascular diseases in aged populations. Endothelial cells (ECs) cover the inner surface of the entire blood vessels, and play critical roles in angiogenesis. During aging, ECs become senescent and senescent ECs have been found to participate in various age-related diseases, including atherosclerosis and diabetes^9^. Cellular senescence impairs the angiogenic capacity of ECs, rendering senescent ECs an attracting therapeutic target for ischemic cardiovascular disease in the aged^10^. In the present study, we explored the effects of spermidine supplementation on the angiogenic capacity of senescent ECs, and revealed the benefits of dietary spermidine on ischemia-induced neovascularization in aged mice.

## Results

### Spermidine supplementation improves the angiogenic capacity of senescent ECs

**Figure 1:**
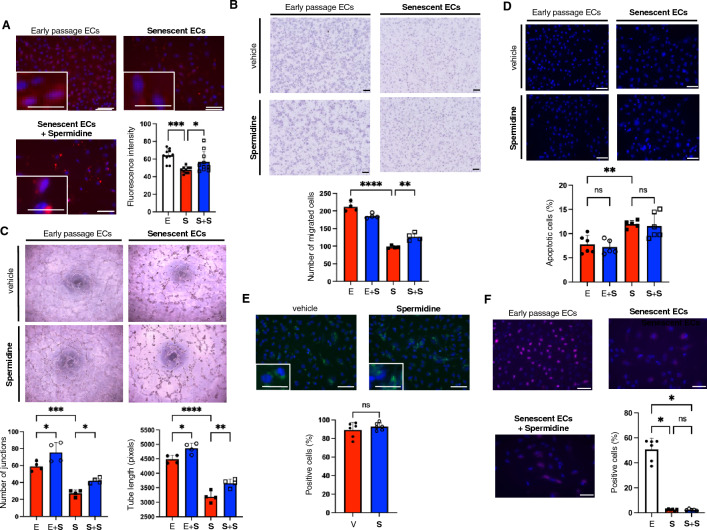
Spermidine supplementation improves angiogenic capacities in senescent ECs. (**A**) Intracellular polyamine staining using PolyamineRED in early passage (E) and replicative senescent ECs (S) with or without spermidine supplementation (+ S). Fluorescence intensity was quantified (n = 10 each). (**B**) Migration capacity was assessed in early passage and replicative senescent ECs using modified Boyden chamber assay (n = 4 each). (**C**) Tube-formation on Matrigel was assessed in early passage and replicative senescent ECs (n = 4 each). (**D**) Apoptosis was induced by serum and growth factor depletion, and apoptotic cells were detected by Hoechst nuclear staining (n = 5–6 each). (**E**) Senescence-associated β-Gal activity was assessed in replicative senescent ECs treated with either vehicle (V) or spermidine (S) using SPiDER-β-Gal staining (n = 6 each). (**F**) Proliferation capacity was assessed by immunocytochemistry for Ki-67. Cells with positive nuclear Ki-67-staining were counted (n = 5–6 each). The difference between the groups was analyzed by one-way ANOVA with Tukey’s post hoc analysis (**A–D**), Kruskal–Wallis test by ranks (**F**), or by two-tailed unpaired Student’s *t*-test (**E**). Data are presented as mean ± S.E. *P < 0.05, **P < 0.01, ***P < 0.001, and ****P < 0.0001. *ns* not significant. Bars: 100 μm.

Replicative senescent ECs were prepared by culturing human ECs for an extended period until cells ceased to proliferate. Cellular senescence was confirmed by reduced proliferation, increased p21 expression, and significant senescence-associated β-Gal activity (Supplementary Fig. 1). We first analyzed whether intracellular polyamine contents in ECs were altered during aging. Intracellular polyamine was significantly reduced in senescent ECs compared to early passage cells (Fig. 1A). Spermidine supplementation in the culture medium increased polyamine contents in senescent ECs to the levels similar to those observed in early passage ECs (Fig. 1A). Since intracellular polyamine in senescent ECs appeared to accumulate in some particular compartments, we decided to assess their potential localization in ER and mitochondria. However, our results indicated that polyamine did not appear to localize and/or accumulate in these particular organelles (Supplementary Fig. 2).

Next, we analyzed the effect of spermidine supplementation on the angiogenic capacity of senescent ECs. Migration and tube-formation capacities were significantly impaired in senescent ECs compared to early passage cells, while apoptosis was enhanced in senescent ECs (Fig. 1B–D). Of note, spermidine supplementation improved the reduced migration and tube-formation capacities in senescent ECs, whereas only the tube-formation capacity was improved in early passage ECs treated with spermidine (Fig. 1B and C). In contrast, spermidine supplementation did not reveal any significant effects on apoptosis in both early passage and senescent ECs (Fig. 1D). Notably, senescence features, such as reduced proliferation and senescence-associated β-Gal activity, were not affected by spermidine supplementation in senescent ECs (Fig. 1E and F). These data strongly suggest that spermidine improves age-related decline in angiogenesis, but fails to rejuvenate senescent ECs.

### Spermidine enhances autophagy in senescent ECs

**Figure 2:**
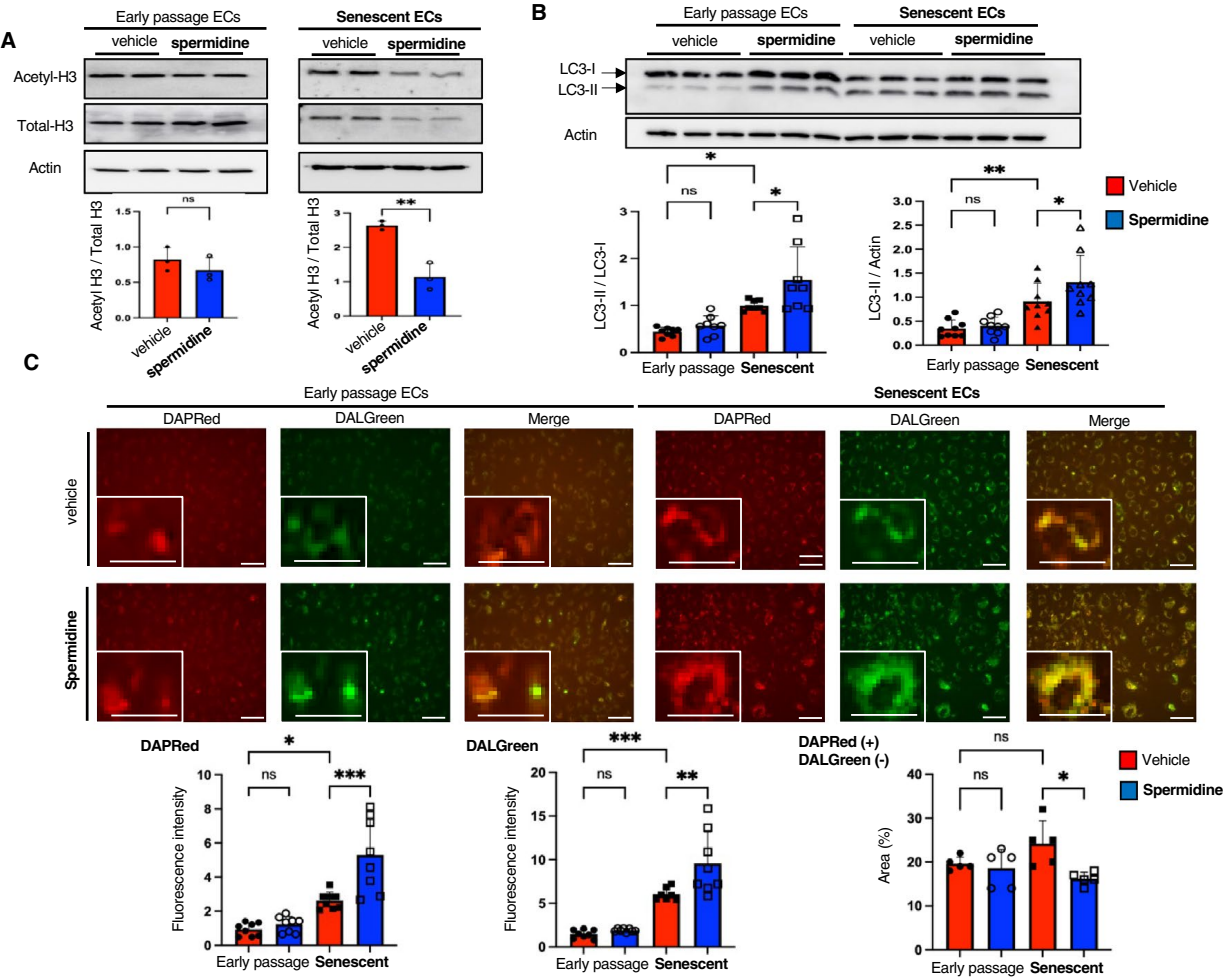
Spermidine supplementation enhances autophagy in senescent ECs. (**A**) Immunoblotting for total- and acetylated histone H3, and actin in early passage and replicative senescent ECs. Acetylated H3 expression levels normalized by total-H3 were quantified (n = 3 each). (**B**) Immunoblotting for LC3 and actin in early passage and replicative senescent ECs. LC3-II expression levels normalized by LC3-I and actin were quantified (n = 8 each). (**C**) Autophagy was assessed using DAPRed (autophagosome) and DALGreen (autolysosome) staining in early passage and replicative senescent ECs in the presence or absence of spermidine supplementation. Fluorescence intensities were quantified (n = 8 each for DAPRed and DALGreen; n = 5 each for DAPRed-positive/DALGreen-negative). The difference between the groups was analyzed by one-way ANOVA with Tukey’s post hoc analysis (**B** and **C**), or by two-tailed unpaired Student’s *t*-test (**A**). Data are presented as mean ± S.E. *P < 0.05, **P < 0.01, and ***P < 0.001. *ns* not significant. Bars: 100 μm.

Because the anti-aging effects of spermidine are largely derived from enhanced autophagy associated with histone deacetylation^11^, we evaluated the histone acetylation in senescent ECs in the presence or absence of spermidine supplementation. Spermidine supplementation was found to reduce histone H3 acetylation in senescent ECs, while no significant effects were detected in early passage cells (Fig. 2A). As mentioned earlier, literature dictates that autophagy is impaired in senescent cells, thus we assessed autophagy in early passage and senescent ECs by using immunoblotting for microtuble-associated protein light chain 3 (LC3). Unexpectedly, autophagosome-associated LC3-II was increased in senescent ECs as compared to that in early passage cells (Fig. 2B). To assess the autophagic flux, cells were treated with bafilomycin A, which inhibits lysosome acidification and autophagosome-lysosome fusion. However, we failed to obtain reliable results probably due to significant cell damage after treatment with bafilomycin A. Consequently, we investigated the autophagic flux by using DAPRed and DALGreen, two hydrophobic dye which can detect autophagosomes and autolysosomes, respectively. Consistent with the increased levels of LC3-II, the obtained DAPRed signals in senescent ECs were also increased compared to early passage cells (Fig. 2C). Surprisingly, DALGreen signals were also enhanced in senescent ECs, indicating high autophagy levels (Fig. 2C). Most of DAPRed-positive autophagosomes colocalized with DALGreen-positive autolysosomes, and the percentage of DALGreen-negative/DAPRed-positive areas were similar in both early passage and senescent ECs (Fig. 2C). These data indicate that autophagic flux was far from disrupted and autophagy was indeed enhanced in the senescent ECs used in our experiments. Given that autophagy is a quality control process that selectively degrades harmful protein aggregates and damaged organelles, the enhanced autophagy detected in senescent ECs might be a compensatory response to maintain cellular quality and/or functions. Furthermore, we identified that the ATP contents in senescent ECs were significantly reduced, a finding that also supports the compensatory enhancement of autophagy in these senescent cells (Supplementary Fig. 3). Of note, spermidine supplementation could further enhance autophagy according to immunoblotting results for LC3-II (Fig. 2B) and by DAPRed and DALGreen staining in senescent ECs (Fig. 2C). Also, spermidine supplementation reduced the DALGreen-negative/DAPRed-positive areas, suggesting accelerated autophagic flux in senescent ECs (Fig. 2C). Considering the crucial role of autophagy in endothelial angiogenic capacity^12–16^, our data strongly suggest that spermidine enhances angiogenic functions in senescent ECs, at least partially, by increasing autophagy.

### Spermidine improves mitochondrial quality in senescent ECs

**Figure 3:**
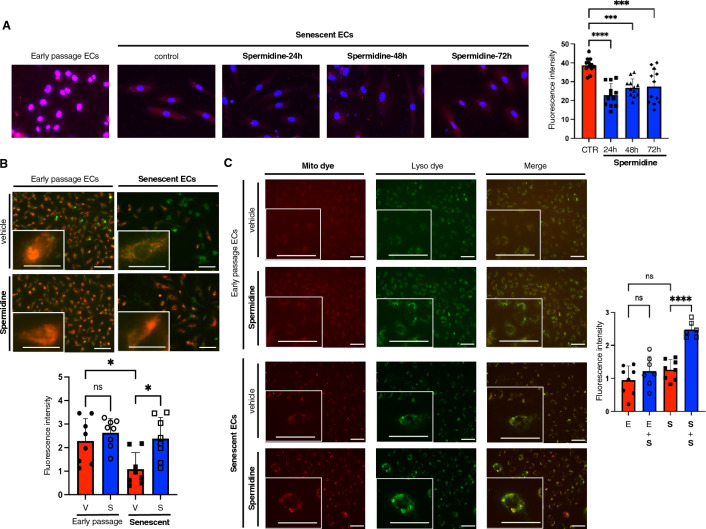
Spermidine supplementation improves mitochondrial quality. (**A**) Mitochondrial superoxide was detected in early passage and replicative senescent ECs using MitoSOX. Senescent ECs were treated with spermidine for 24, 48, or 72 h. Fluorescence intensity was quantified (n = 12 each). (**B**) Mitochondrial membrane potential was assessed by using TMRM staining. Early passage and replicative senescent ECs were treated with either vehicle (V) or spermidine (S) for 48 h before the assay. TMRM fluorescence (red) intensity, which correlates mitochondrial membrane potential, was measured, and normalized with MitoTracker fluorescence (green) intensity (n = 8 each). (**C**) Mitophagy was assessed using Mito dye that accumulates in mitochondria and is activated at acidic ph. Well-colocalization of Mito dye with lyosome (Lyso dye) was confirmed. Early passage (E) and replicative senescent ECs (S) were treated with either vehicle (V) or spermidine (S) for 48 h before the assay. Fluorescence intensity for Mito dye was quantified (n = 7–8 each). The difference between the groups was analyzed by one-way ANOVA with Tukey’s post hoc analysis. Data are presented as mean ± S.E. *P < 0.05, ***P < 0.001, and ****P < 0.0001. *ns* not significant. Bars: 100 μm.

Since mitochondrial dysfunction is causally involved in cellular senescence^17^, we explored the effects of spermidine on mitochondrial quality in senescent ECs. MitoSOX fluorogenic dyes, which specifically target mitochondria, showed that mitochondrial reactive oxygen species (ROS) levels increased, and a significant decrease in their membrane potential in senescent ECs compared to early passage cells was identified by using Tetramethylrhodamine, methyl ester (TMRM) and JC-1 staining (Fig. 3A,B, and Supplementary Fig. 4). Despite the fact that mitochondrial staining was barely detectable in early passage cells cells, we detected unspecific accumulation of MitoSOX in the nuclei of early passage cells, as shown in Fig. 3A. Notably, spermidine supplementation reduced mitochondrial superoxide and increased the mitochondrial membrane potential in senescent ECs (Fig. 3A,B, and Supplementary Fig. 4). Our findings clearly suggest that spermidine could enhance the autophagy in senescent ECs. We therefore presumed that spermidine could probably improve mitochondrial quality via enhanced mitophagy^18^. As shown in Fig. 3C, mitophagy assessment revealed that spermidine supplementation enhanced the mitophagy in senescent ECs.

### Spermidine improves mitochondrial quality via enhanced autophagy

**Figure 4:**
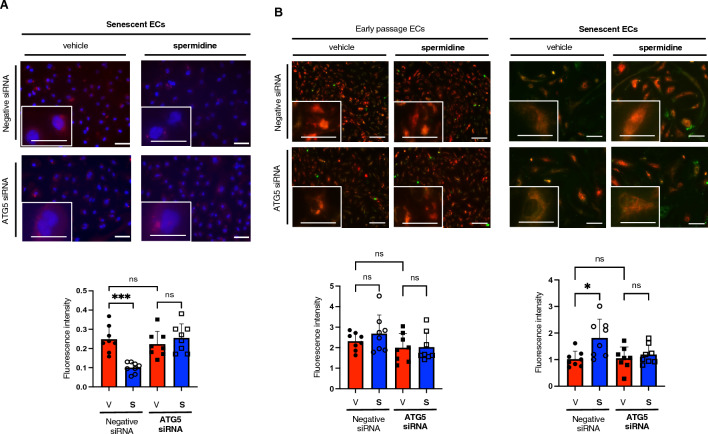
Spermidine improves the mitochondrial quality via enhancing autophagy. (**A**) Mitochondrial superoxide was detected using MitoSOX. Senescent ECs were treated with either vehicle (V) or spermidine (S) for 48 h before the assay. Cells were transfected with either ATG5 or negative siRNA. Fluorescence intensity was quantified (n = 8 each). (**B**) Mitochondrial membrane potential was assessed in early passage and replicative senescent ECs using TMRM. Mitochondria was stained using MitoTracker. Cells were treated with either vehicle (V) or spermidine (S) for 48 h before the assay. TMRM fluorescence intensities normalized with MitoTracker fluorescence intensities were shown (n = 8 each).

To confirm the critical role of autophagy in improving mitochondrial quality in senescent ECs supplemented with spermidine, autophagy was inhibited by gene silencing of ATG5^19^. ECs transfected with ATG5 siRNA showed significant reduction of ATG5 expression both in young and senescent cells, and spermidine supplementation was not found to affect the ATG5 silencing (Supplementary Fig. 5). Of note, ATG5 silencing completely abolished the reduction in mitochondrial ROS and increased mitochondrial membrane potential in senescent ECs supplemented with spermidine (Fig. 4). These data strongly suggest that spermidine improves the mitochondrial quality in senescent ECs largely by enhancing autophagy.

### Dietary spermidine improves the declined neovascularization in aged mice

**Figure 5:**
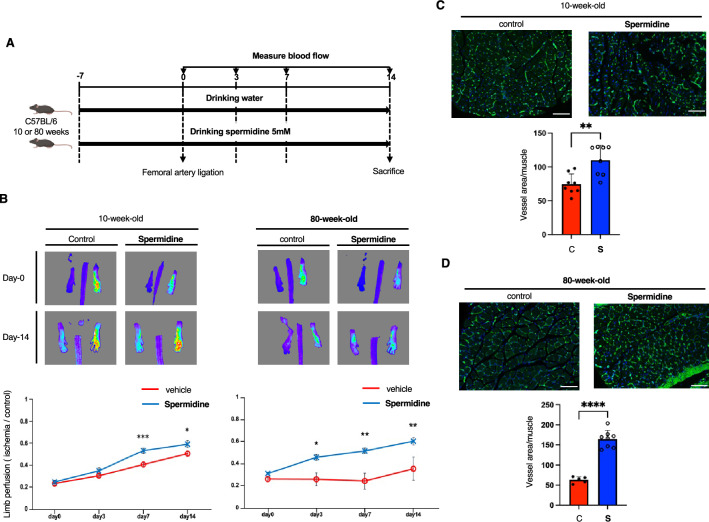
Dietary spermidine improves ischemia-induced neovascularization in aged mice. (**A**) Experimental scheme was shown. (**B**) Blood flow in ischemic limbs was analyzed by laser Doppler flowmetry at Day-0, 3, 7, and 14 post surgery (n = 8 each for 10-week-old mice; n = 8 for 80-week-old mice treated with spermidine; n = 8 at Day-0, n = 6 at Day-3, and n = 5 at Day-7 and -14 for 80-week-old control mice). (**C**,**D**) Blood vessels in ischemic muscles of young (**C**) and aged (**D**) mice were stained. Ischemic muscles were dissected from control or spermidine-treated mice at Day-14 post surgery. Capillary density was quantified (n = 8 each for **C**; n = 5 for control and n = 8 for spermidine group for **D**). The difference between the groups was analyzed by two-tailed unpaired Student’s *t*-test (**B** and **D**) or Mann–Whitney *U*-test (**C**). Data are presented as mean ± S.E. *P < 0.05, **P < 0.01, ***P < 0.001, and ****P < 0.0001. Bars: 100 μm.

We finally analyzed the ischemia-induced neovascularization in young (10-week-old) and aged (80-week-old) mice. Dietary spermidine was given through drinking water, and the blood flow in ischemic limbs was analyzed by laser-Doppler flowmetry at days 0, 3, 7, and 14 days post surgery (Fig. 5A). Blood flow recovery in ischemic limbs was substantially reduced in aged mice compared to young mice (Fig. 5B). Of note, dietary spermidine significantly improved the blood flow recovery in ischemic limbs in aged mice, while only minimal effects were observed in young mice (Fig. 5B). Consistent with the above findings, the blood vessel density in ischemic muscles was increased by dietary spermidine, and the effects were more robust in aged mice (Fig. 5C and D).

To assess the role of autophagy in the enhanced angiogenesis in mice supplemented with spermidine, the LC3 expression in ischemic muscle was analyzed. LC3-II expression was significantly increased in the ischemic muscles of aged mice supplemented with spermidine, whereas no significant effects were detected in young mice (Supplementary Fig. 6A). To further assess autophagy in ECs, we performed immunohistochemistry for LC3 using a section of the ischemic muscle. Both in young and aged mice supplemented with spermidine demonstrated a significantly increased number of ECs positive for LC3-staining (Supplementary Fig. 6B). These data strongly suggest that spermidine supplementation could enhance ischemia-induced angiogenesis, at least partially, by enhancing autophagy in ECs.

## Discussion

Spermidine is a naturally occurring polyamine that has shown substantial evidence of anti-aging properties^3^. The aging process has been found to reduce endogenous spermidine concentrations, a phenomenon that is not observed in centenarians, which show relatively high concentration of spermidine^3^. Dietary supplementation of spermidine extends the lifespan of various organisms, including mammals^1^. Furthermore, dietary spermidine improves cognitive functions and demonstrates cardioprotective effects, indicating its beneficial role in extending a healthy lifespan^6,7^. Notably, these beneficial effects of spermidine tend to disappear in the absence of autophagy, suggesting that autophagy enhancement is the central mechanism in the effects of spermidine. In humans, high levels of spermidine intake correlate with improved cognitive performance, reduced blood pressure, lower incidence of cardiovascular disease, and lower all-cause mortality^20^. Consequently, these data have shown dietary spermidine as a potential pharmacological anti-aging intervention, although recent clinical trials have failed to confirm its beneficial effects on maintaining memory performance^21,22^.

Cellular senescence plays a fundamental role in the aging process^23^. Accumulation of senescent cells is harmful for nearby healthy cells via senescence-associated secretory phenotypes, resulting in organ dysfunction^24,25^. Selective elimination of senescent cells has shown beneficial effects on age-related diseases, and notably, extended the lifespan of mice^26–28^. It has been reported that spermidine prevents cellular senescence, which may even reversed in particular types of cells^29,30^. Despite the fact that several reports have described the effect of spermidine on EC functions^31^, little is known about the role of dietary spermidine in the age-related decline of EC functions. EC senescence is crucially involved in the onset and progression of age-related diseases. Moreover, it has been revealed that vascular aging is a hierarchically high driver of overall organismal aging due to failure to maintain adequate microvascular density^32^. These data urged us to explore the effects of spermidine on EC angiogenic functions, especially in senescent cells.

The present study demonstrated that spermidine supplementation could improve the angiogenic capacity of senescent ECs associated with enhanced autophagy and mitophagy. The molecular mechanisms through which spermidine enhances autophagy have been thoroughly analyzed^3,11,33^, thus we presumed that theses mechanisms are largely preserved among various cell types as we detected the reduced histone H3 acetylation in senescent ECs treated with spermidine. However, further analysis is required to clarify the detailed mechanisms underlying the enhanced autophagy in ECs supplemented with spermidine. Since senescence features remained largely unaffected, the beneficial effects of spermidine are probably independent of rejuvenation. Unexpectedly, the replicative senescent ECs that we used in the present experiments exhibited augmented autophagy as opposed to young early passage cells. Although senescent cells often show reduced autophagic capacity, cells undergoing senescence may maintain cellular functions by enhancing autophagy at certain time point as a compensatory mechanism. The lower ATP contents found in senescent ECs clearly suggest that the enhanced autophagy detected in senescent ECs might be a compensatory response. In addition, activation of autophagy during senescence has also been reported^34,35^. Spermidine supplementation further enhanced autophagy preferentially in senescent ECs. Considering the crucial role of autophagy in endothelial angiogenic capacity, autophagy enhancement and the consequent improvement in mitochondrial quality are probably the major mechanism for the observed proangiogenic effects of spermidine in senescent ECs; however, further analysis is required to validate these underlying mechanisms.

There are several limitations in the current study. First, we used human umbilical vein ECs (HUVECs) for all the in vitro experiments. HUVECs are widely used for the analysis of endothelial functions due to the highly consistent and stable cellular characteristics regardless of the source of the cells (i.e. donors). However, ECs have unique features depending on the vascular beds, and therefore, analysis using microvascular ECs, which probably play a central role in ischemia-induced angiogenesis, is more suitable for assessing angiogenic functions. Furthermore, the significantly beneficial effect of spermidine supplementation in ischemia-induced neovascularization in aged mice was shown; however, this effect was less prominent in young mice. In these experiments, we used 10-week-old mice as the (young) control. Although adulthood is biologically defined as the age at which sexual maturity is attained (~ 10 weeks-old for mice)^36^, there have been several reports on mice stating that this rapid growth continues up to three months in mice. Therefore, the control mice we used may not have been fully matured, affecting our experimental results.

Since angiogenic capacity is positively associated with tumor growth, improved angiogenic capacity in senescent ECs could promote tumor growth in aged populations. In addition, it has been suggested that polyamines might have pro-carcinogenic properties because of their proliferation-enhancing and cytoprotective effects^2^. However, spermidine has also been associated with anti-carcinogenesis effects^2^; therefore, the true role of polyamine in cancer progression remains controversial. Careful consideration and in-depth investigation are required before dietary spermidine supplementation can be broadly recommended as an anti-aging intervention. Nevertheless, the lifespan-extending effects of spermidine strongly suggest its gross beneficial properties as an anti-aging compound. Our data provide previously undescribed proangiogenic effects of spermidine, suggesting its therapeutic potential against ischemic diseases, especially in the aged.

## Materials and methods

### Materials

Spermidine was purchased from Nacalai Tesque (#32108-04). Antibody for LC3 (#0231-100) was obtained from nanoTools. Antibodies for total Histon H3 (#4499), acetylated Histone H3 (#9649), and p21 (#2947) were obtained from Cell Signaling Technology. Antibody for β-actin (#A2228) was obtained from Sigma. Human umbilical vein endothelial cells (HUVECs) were obtained from LIFELINE Cell Technology.

### Cell culture

Cells were regularly cultured in the CO_2_ cell culture incubator (at 37 °C under 5% CO_2_ levels). Replicative senescent ECs were prepared by culturing cells for extensive period of time. HUVECs were cultured in HuMedia-EG2 medium (Kurabo #KE-2150S), and regularly passaged at 1:4 ratio when reached sub-confluent. Cells were passaged until they did not show obvious proliferation with enlarged and flattened morphology (usually passage 18–20). Cellular senescence was confirmed by enhanced senescence-associated β-GAL activity, reduced proliferation, and increased expression of senescence-associated genes such as p21. P3 HUVECs were regularly used as young early passage ECs.

### Immunoblotting

Cells were lysed in RIPA buffer containing protease inhibitor cocktail (Sigma #P8340). After measurements of concentration using DC protein assay kit (BioRad #500112), the same amount of proteins were run on SDS-PAGE gel, followed by transfer onto nitrocellulose membrane. The membrane was incubated with antibody for LC3 (1:1000), acetyl-Histone H3 (Lys9) (1:1000), Histone H3 (1:1000), p21 (1:1000), or β-actin (1:2000) at 4 °C for overnight, following blocking with 5% skim milk. After washing with TBS-T, the membrane was incubated with HRP-labelled appropriate secondary antibody (1:2000), followed by signal detection using ChemiDoc Touch MP (BioRad). Results were confirmed by at least two independent experiments.

### Immunocytochemistry

Cells were fixed using 4% PFA, followed by permeabilization with 0.1% Triton-X. After blocking with 10% normal donkey serum, cells were incubated with anti-Ki-67 antibody (1:200) at 4 °C for overnight. After washing with PBS, cells were incubated with fluorescence-labelled secondary antibody (1:500). Subsequently, cells were mounted with antifade mounting medium with DAPI (Vector laboratory #H-1200), followed by analysis under all-in-one fluorescence microscopy (Keyence).

### Detection of intracellular polyamine

Intracellular polyamines were detected using PolyamineRED (Funakoshi #FDV-0020) by following the manufacturer’s recommendations. Early passage and replicative senescent ECs plated on 48-well plate were cultured in the presence or absence of 150 μM spermidine for 48 h. Cells were incubated with 20 μM PolyamineRED and 10 μg/ml Hoechst 33342 (Thermo Fisher #H1399) for 1 h at 37 °C in the CO_2_ incubator. After washing with PBS for three times, cells were fixed with 4% PFA for 10 min, followed by observation under fluorescence microscopy (Keyence). Results were confirmed by at lease two independent experiments.

### Determination of mitochondrial ROS

Mitochondrial superoxide was detected using the fluorescent MitoSOX Red probe (Invitrogen #M36008). Cells were incubated in Hank’s Balanced Salt solution (HBSS) containing 5 μM MitoSOX-Red for 10 min at 37 °C in the CO_2_ incubator. After washing with PBS, cells were observed under fluorescence microscopy (Keyence). The fluorescence intensities of randomly selected cells were analyzed by using the Image-J software. Results were confirmed by at least two independent experiments.

### Mitochondrial membrane potential assay

The mitochondrial membrane potential was assessed using the JC-1 MitoMP Detection Kit (DOJINDO #MT09) and Image-iT TMRM reagent (Thermo) as the manufacturer recommends. Early passage and replicative senescent ECs were incubated with TMRM reagent (1:1000) and MitoTracker Green (Thermo, 1:5000) for 30 min at 37 °C in the CO_2_ incubator, followed by washing with PBS for 3 times. Subsequently cells were observed under fluorescent microscopy (Keyence). Some cells were treated with 150 μM spermidine for 48 h prior to the assay. Fluorescence intensities for TMRM and MitoTracker were measured using the image-J software, and the mitochondrial membrane potential was assessed by the TMRM fluorescence intensity normalized by MitoTracker intensity. For JC-1 assay, Early passage and replicative senescent ECs were incubated with 2 μM JC-1 for 1 h at 37 °C in the CO_2_ incubator. After washing with HBSS twice, Imaging Buffer solution was added, followed by analysis of cells under fluorescence microscopy (Keyence). When the mitochondrial membrane potential is high, JC-1 forms red fluorescent “J-aggregates”, while JC-1 dye remain as monomer, exhibiting green fluorescence in mitochondria with low membrane potential. The mitochondrial membrane potential was assessed by the ratio of red/green fluorescence intensity. Results were confirmed by at least two independent experiments.

### Gene silencing for ATG5

ECs were transfected with 100 nM ATG5 (SignalSilence Atg5 siRNA, Cell Signaling Technology #6345) or negative siRNA (siRNA Universal Negative control#1, Sigma #SIC001) using Lipofectamine RNAiMAX reagent (Invitrogen #137878075). Cells were used for each assay 48 h after siRNA transfection.

### SPiDER-β-Gal staining

Senescence-associated β-Gal activity was detected by SPiDER β-Gal (DOJINDO #SG02) by following the manufacturer’s recommendation. Early passage and replicative senescent ECs plated on 48-well plate were washed with HBSS, followed by incubation with HBSS containing 50 nM Bafilomycin A1 in the CO_2_ incubator for 1 h. Cells were then incubated with SPiDER-β-Gal solution and 10 μg/ml Hoechst 33342 for 30 min, followed by washing with HBSS, and then observed under fluorescence microscopy (Keyence).

### Tube formation assay

In vitro angiogenesis analysis was performed in 96-well plates coated with 50 μL of Matrigel (Corning #356234). Seven × 10^3^ HUVECs were plated on Matrigel and incubated for 3–6 h. Images were taken using an inverted microscope (OLYMPUS IX71), and the tube length and the number of junctions were measured in 4 independent fields. Some cells were treated with 150 μM spermidine for 48 h prior to the assay. Results were confirmed by at least two independent experiments.

### Migration assay

Migration capacity was analyzed by modified Boyden chamber assay using the Transwell membrane Insert with 8.0 μm (Corning #3422). ECs were resuspended in migration buffer (DMEM containing 1% BSA) and 8.4 × 10^4^ cells were placed in the insert. Subsequently, the inserts were put on 12-well plate where DMEM containing 2% fetal bovine serum was added. After 4 h incubation, cells remaining in the top chamber was scraped off using cotton swab, and the migrated cells were stained with Mayer’s Hematoxylin Solution (Wako #131-09665), following fixation with methanol. Some cells were treated with 150 μM spermidine for 48 h prior to the assay.

### Apoptosis assay

ECs plated on 48-well plate were treated with 150 μM spermidine for 48 h. After the treatment, apoptosis was induced by serum and growth factor depletion for 24 h. Nuclei were stained with Hoechst 33342, and cells with condensed and/or fragmented nuclei were counted as apoptotic cells. Results were confirmed by at least two independent experiments.

### ATP measurements

Cellular ATP contents were measured by using ATP Assay Kit-Luminescence (DOJINDO #A550) as the manufacturer recommends. Measurements were performed in duplicate, and luminescence generated by ATP was measured using the TECAN Infinite 200 Pro Microplate reader.

### Animals

All experimental protocols were approved by the Ethics Review Committee for Animal Experimentation of Kyoto Prefectural University of Medicine (#M2021-537). Animal experiments were performed in compliance with ARRIVE (Animal Research: Reporting of In Vivo Experiments) guidelines, and all experiments were performed in accordance with relevant guidelines and regulations. Mice were housed in designated cages of sufficient size (1–3 mice in one cage) in animal facility in which the temperature and humidity are regulated at 〜23 °C and 〜60%, respectively. Mice were maintained under a 12-h light/12-h dark cycle, and fed chow with ad libitum access to water and food.

### Hind-limb ischemia model

Male C56BL/6 J mice at the age of 10 weeks or 80 weeks old were used for hind-limb ischemia model. Mice were anesthetized using 2% isoflurane, and underwent surgically induced unilateral hindlimb ischemia. After ligation, the femoral artery was removed from its origin just above the inguinal ligament to its bifurcation at the origin of the saphenous and popliteal arteries (Day-0). Limb blood flow was determined using a laser Doppler blood flow (LDBF) analyzer, OMEGAZONE-1 (OMEGAWAVE) at Day-0, 3, 7, and 14. LDBF analyses were performed on the soles of mice on the indicated postoperative days. Blood flow is shown as changes in the laser frequency using different color pixels. Quantitative analysis of blood flow was expressed as the ratio of left (ischemic) to right (nonischemic) LDBF. At Day-14, ischemic muscles were dissected, and subjected to histological analysis. Dietary spermidine was supplemented through drinking water containing 5 mM spermidine one week before the operation.

### Measurements of capillary density

Ischemic muscles dissected at Day-14 were fixed with 4% PFA, followed by embedding in Tissue-Tek O.C.T. Compound (Sakura Finetek) and flash frozen. Frozen sections were stained with isolectin GS-IB_4_ (Invitrogen #I21411), followed by mounting with antifade mounting medium with DAPI (Vector laboratory #H-1200). Sections were then analyzed under fluorescence microscopy (Keyence). Capillary density was expressed as the isolectin-positive area normalized by muscles numbers. Three randomly selected fields from transverse sections were analyzed per animal.

### Immunohistochemistry

Frozen sections of ischemic muscles dissected at Day-14 were incubated with anti-LC3 antibody and isolectin GS-IB_4_ for overnight at 4 °C, following the blocking with 5% donkey serum. After washing with PBS, sections were incubated with secondary antibody conjugated with Alexa Fluor 488 for 1 h at room temperature, followed by washing with PBS and mounting with antifade mounting medium with DAPI. Sections were then analyzed under fluorescence microscopy (Keyence). The number of isolectin-positive ECs that are positive for LC3 was counted in 3–5 randomly selected fields for each section.

### Statistical analysis

All data are presented as mean ± S.E. Data normality was assessed using Shapiro–Wilk test. The difference between 2 groups was analyzed using two-tailed unpaired Student’s *t*-test, while differences between groups more than 3 were analyzed using one-way ANOVA with Tukey’s post hoc test as described in each figure legend. In case the data normality was not shown, the difference between groups was analyzed using non parametric Mann–Whitney *U*-test for comparison between 2 groups or Kruskal–Wallis test by ranks for comparison between groups more than 3. *P* < 0.05 is considered as statistically significant.

## Supplementary Information

Supplementary Information.

Supplementary Figures.

## Data Availability

All data generated or analyzed during this study are included in this published article and its supplementary information files.

## References

[1] de Cabo R, Carmona-Gutierrez D, Bernier M, Hall MN, Madeo F (2014). The search for antiaging interventions: From elixirs to fasting regimens. Cell.

[2] Madeo F, Eisenberg T, Pietrocola F, Kroemer G (2018). Spermidine in health and disease. Science.

[3] Madeo F, Bauer MA, Carmona-Gutierrez D, Kroemer G (2019). Spermidine: A physiological autophagy inducer acting as an anti-aging vitamin in humans?. Autophagy.

[4] Pucciarelli S (2012). Spermidine and spermine are enriched in whole blood of nona/centenarians. Rejuvenation Res..

[5] Eisenberg T (2009). Induction of autophagy by spermidine promotes longevity. Nat. Cell Biol..

[6] Schroeder S (2021). Dietary spermidine improves cognitive function. Cell Rep..

[7] Eisenberg T (2016). Cardioprotection and lifespan extension by the natural polyamine spermidine. Nat. Med..

[8] Hansen M, Rubinsztein DC, Walker DW (2018). Autophagy as a promoter of longevity: Insights from model organisms. Nat. Rev. Mol. Cell Biol..

[9] Honda S (2021). Cellular senescence promotes endothelial activation through epigenetic alteration, and consequently accelerates atherosclerosis. Sci. Rep..

[10] Uraoka M (2011). Loss of bcl-2 during the senescence exacerbates the impaired angiogenic functions in endothelial cells by deteriorating the mitochondrial redox state. Hypertension.

[11] Pietrocola F (2015). Spermidine induces autophagy by inhibiting the acetyltransferase EP300. Cell Death Differ..

[12] Mameli E, Martello A, Caporali A (2022). Autophagy at the interface of endothelial cell homeostasis and vascular disease. FEBS J..

[13] Schaaf MB, Houbaert D, Mece O, Agostinis P (2019). Autophagy in endothelial cells and tumor angiogenesis. Cell Death Differ..

[14] Vion AC (2017). Autophagy is required for endothelial cell alignment and atheroprotection under physiological blood flow. Proc. Natl. Acad. Sci. U. S. A..

[15] Sprott D (2019). Endothelial-specific deficiency of ATG5 (autophagy protein 5) attenuates ischemia-related angiogenesis. Arterioscler. Thromb. Vasc. Biol..

[16] Menghini R (2014). MiR-216a: A link between endothelial dysfunction and autophagy. Cell Death Dis..

[17] Martini H, Passos JF (2022). Cellular senescence: All roads lead to mitochondria. FEBS J..

[18] Pickles S, Vigie P, Youle RJ (2018). Mitophagy and quality control mechanisms in mitochondrial maintenance. Curr. Biol..

[19] Ye X, Zhou XJ, Zhang H (2018). Exploring the role of autophagy-related gene 5 (ATG5) yields important insights into autophagy in autoimmune/autoinflammatory diseases. Front. Immunol..

[20] Kiechl S (2018). Higher spermidine intake is linked to lower mortality: A prospective population-based study. Am. J. Clin. Nutr..

[21] Schwarz C (2018). Safety and tolerability of spermidine supplementation in mice and older adults with subjective cognitive decline. Aging (Albany NY).

[22] Schwarz C (2022). Effects of spermidine supplementation on cognition and biomarkers in older adults with subjective cognitive decline: A randomized clinical trial. JAMA Netw. Open.

[23] van Deursen JM (2014). The role of senescent cells in ageing. Nature.

[24] Rodier F, Campisi J (2011). Four faces of cellular senescence. J. Cell Biol..

[25] Tchkonia T, Zhu Y, van Deursen J, Campisi J, Kirkland JL (2013). Cellular senescence and the senescent secretory phenotype: Therapeutic opportunities. J. Clin. Invest..

[26] Baker DJ (2011). Clearance of p16Ink4a-positive senescent cells delays ageing-associated disorders. Nature.

[27] Baker DJ (2016). Naturally occurring p16(Ink4a)-positive cells shorten healthy lifespan. Nature.

[28] Xu M (2018). Senolytics improve physical function and increase lifespan in old age. Nat. Med..

[29] Carnevale R (2021). Beneficial effects of a combination of natural product activators of autophagy on endothelial cells and platelets. Br. J. Pharmacol..

[30] Zhang H (2019). Polyamines control eIF5A hypusination, TFEB translation, and autophagy to reverse B cell senescence. Mol. Cell.

[31] Camprecios G (2021). Spermidine supplementation protects the liver endothelium from liver damage in mice. Nutrients.

[32] Grunewald M (2021). Counteracting age-related VEGF signaling insufficiency promotes healthy aging and extends life span. Science.

[33] Minois N (2014). Molecular basis of the 'anti-aging' effect of spermidine and other natural polyamines—A mini-review. Gerontology.

[34] Young AR (2009). Autophagy mediates the mitotic senescence transition. Genes Dev..

[35] Kwon Y, Kim JW, Jeoung JA, Kim MS, Kang C (2017). Autophagy is pro-senescence when seen in close-up, but anti-senescence in long-shot. Mol. Cells.

[36] Dutta S, Sengupta P (2016). Men and mice: Relating their ages. Life Sci..

