# Supplementary material for: Spermidine improves angiogenic capacity of senescent endothelial cells, and enhances ischemia-induced neovascularization in aged mice
Source: Sci Rep. 2023 May 23;13:8338. doi: 10.1038/s41598-023-35447-3 (PMC10205711; doi:10.1038/s41598-023-35447-3)
Supplement: Supplementary file 2 — Supplementary Figures. [file 41598_2023_35447_MOESM2_ESM.pdf]

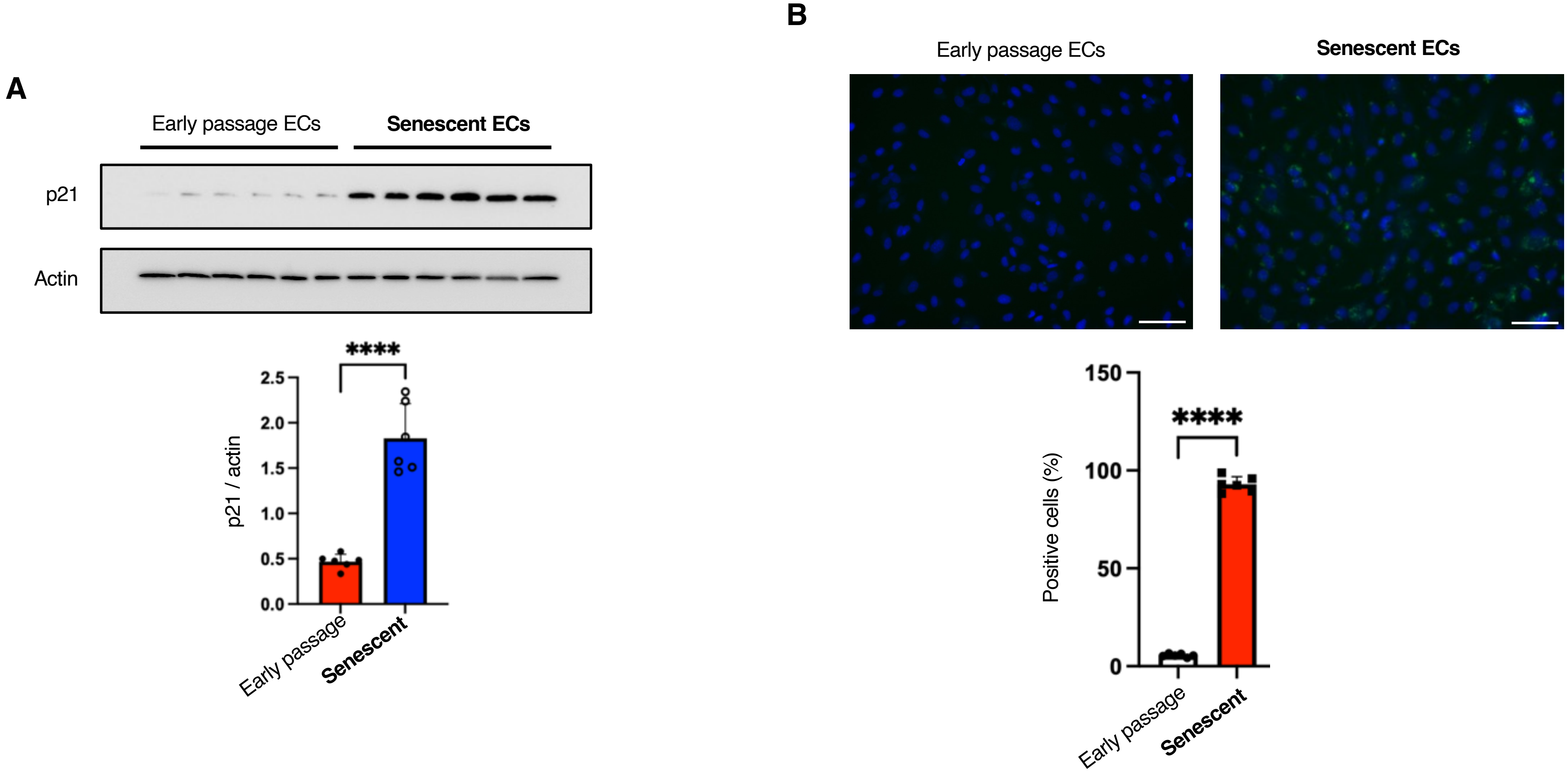

Supplementary Figure-1.
(**A**) Immunoblotting for p21 and actin in early passage and replicative senescent ECs (n = 6 each).
(**B**) SPiDER-β-Gal staining in early passage and replicative senescent ECs. Cells with positive staining were counted (n = 6 each). The difference between the groups was analyzed by two-tailed unpaired Student's *t*-test. Data are presented as mean ± S.E. $^{****}P < 0.0001$. Bars: 100 μm.

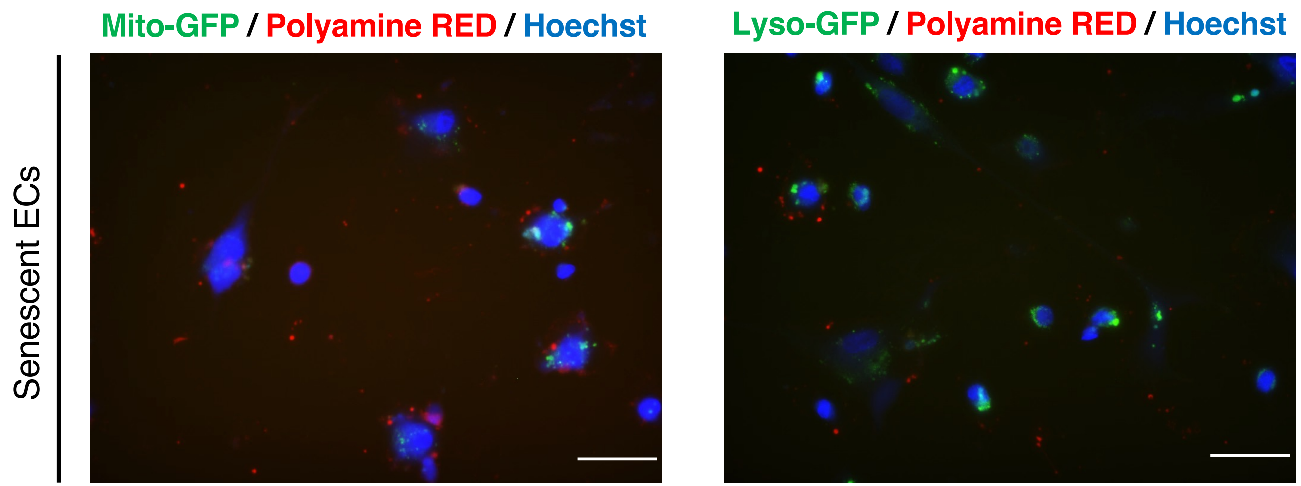

Supplementary Figure-2.
Fluorescent staining for mitochondria (Mito-GFP) and lysosome (Lyso-GFP) in conjunction with polyamineRED staining. Bars: 100 μm.

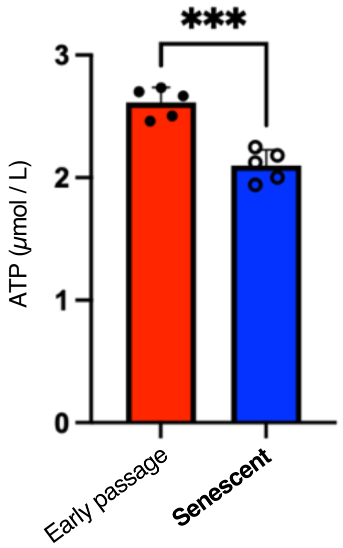

Supplementary Figure-3.
Cellular ATP contents were analyzed in early passage and replicative senescent ECs (n = 6 each). The difference between the groups was analyzed by two-tailed unpaired Student's *t*-test. Data are presented as mean ± S.E. $^{***}P < 0.001$.

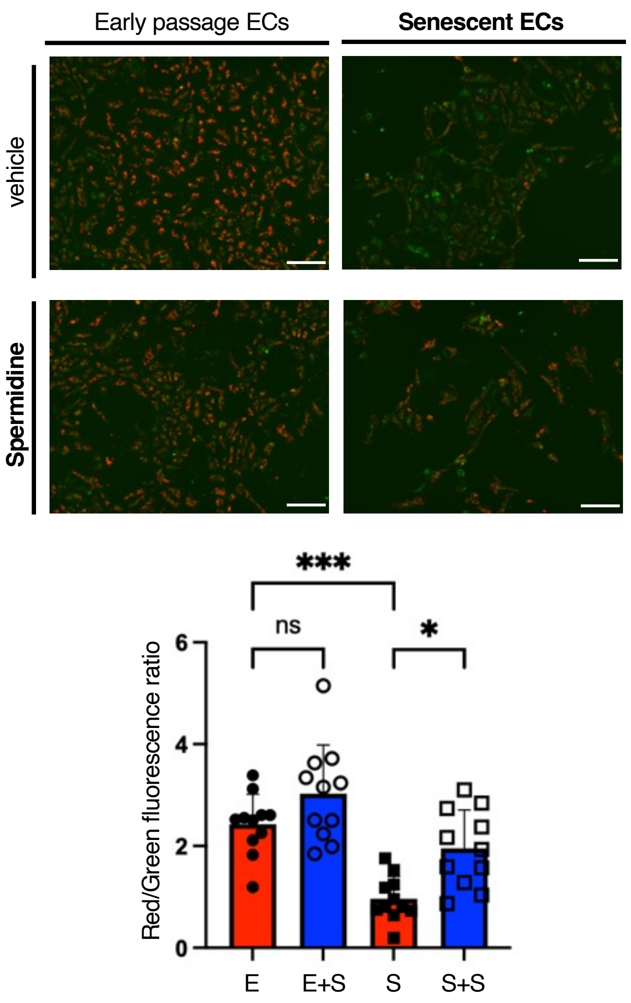

Supplementary Figure-4.
Mitochondrial membrane potential was assessed by JC-1 staining. Red/Green fluorescence ratio, which correlates mitochondrial membrane potential, was measured (n =11 each). The difference between the groups was analyzed by one-way ANOVA with Tukey's post hoc analysis. Data are presented as mean ± S.E. *$P < 0.05$ and ***$P < 0.001$. ns; not significant. Bars: 100 μm.

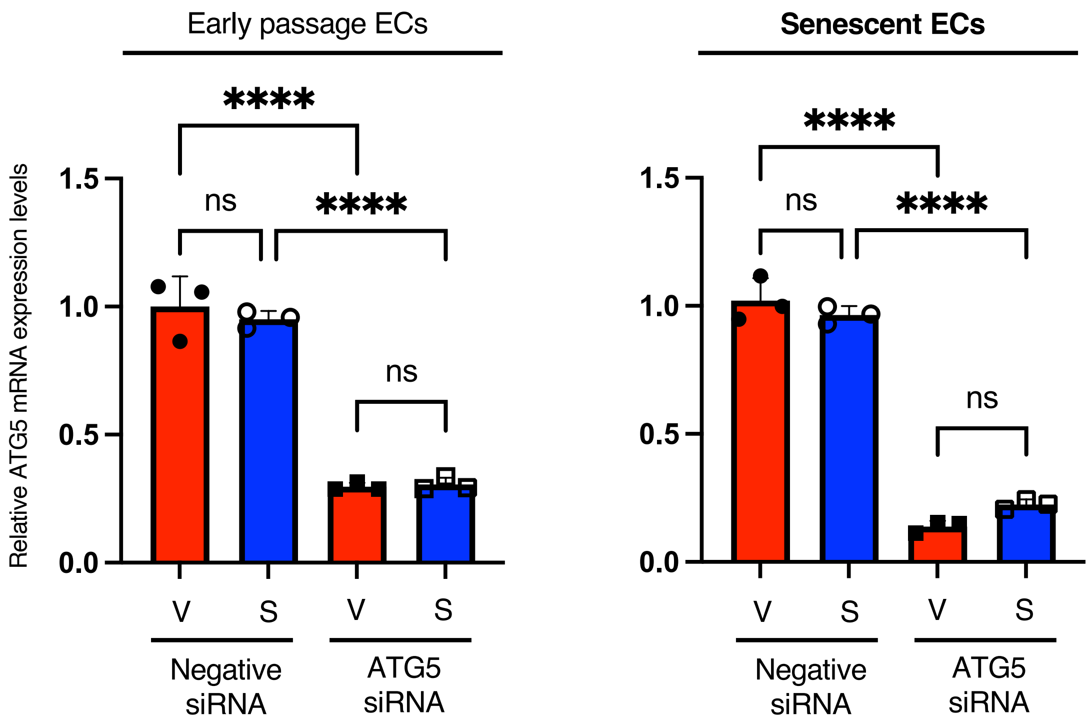

Supplementary Figure-5.
Quantitative PCR for ATG5 mRNA in early passage or senescent ECs treated with either vehicle or spermidine. Some cells were transfected with ATG5 siRNA, while the others were transfected with negative siRNA (n = 3 each). The difference between the groups was analyzed by on-way ANOVA with Tukey's post hoc test. Data are presented as mean ± S.E. ****$P < 0.0001$. ns; not significant.

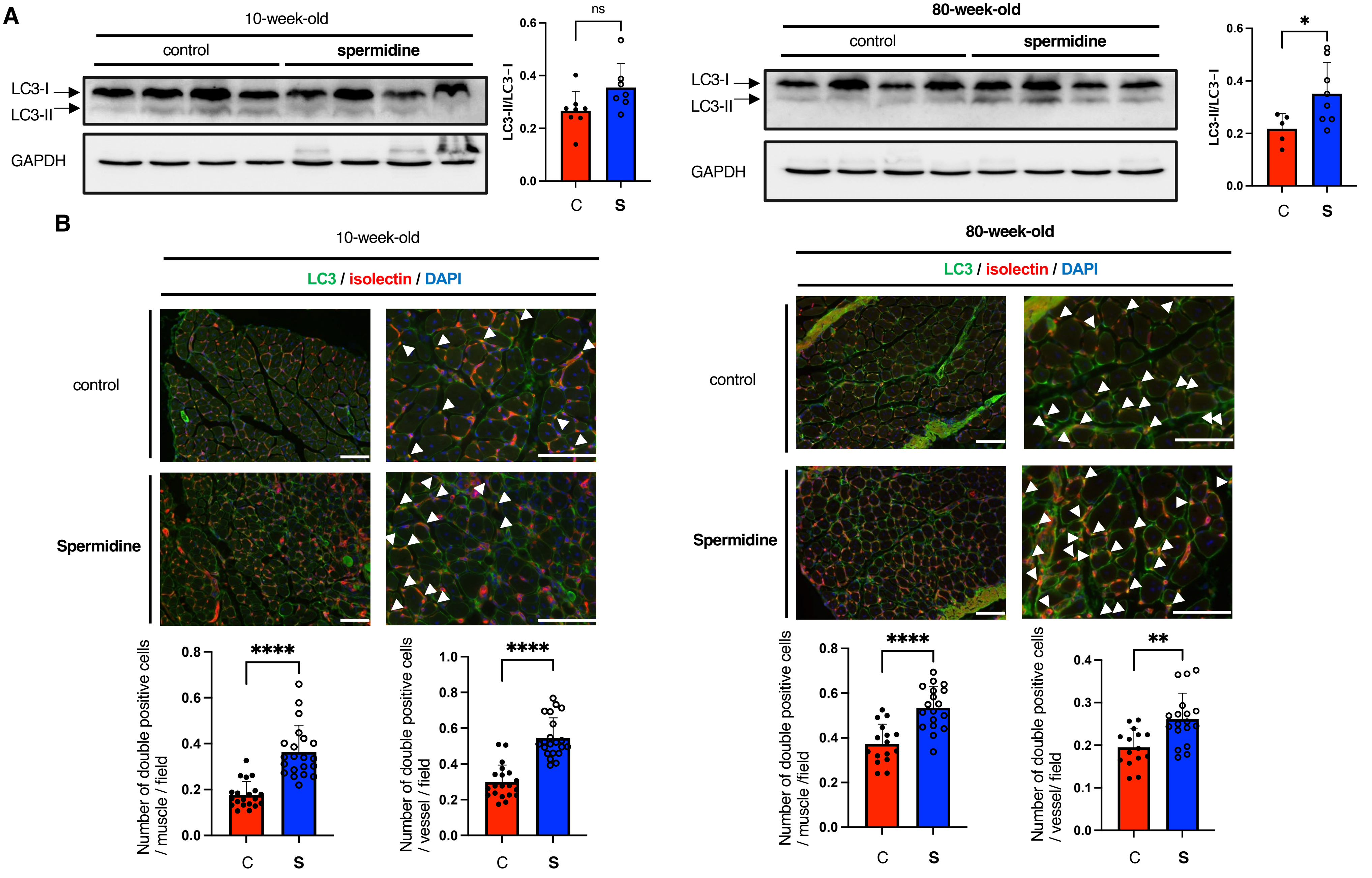

Supplementary Figure-6.

(A) Immunoblotting for LC-3 and GAPDH in ischemic muscle isolated from control or spermidine-treated mice (n = 8 for young control group; n = 7 for young spermidine group; n = 5 for aged control group; n = 8 for aged spermidine group).

(B) Immunohistochemistry for LC3 and isolectin-staining in ischemic muscles. Cells double positive for LC3 and isolectin (arrowhead) were counted and normalized by the number of either muscles or isolectin-positive vessels (n = 20 each). The difference between the groups was analyzed by two-tailed unpaired Student's *t*-test. Data are presented as mean ± S.E. $^{*}P < 0.05$, $^{**}P < 0.01$, and $^{****}P < 0.0001$. ns; not significant.

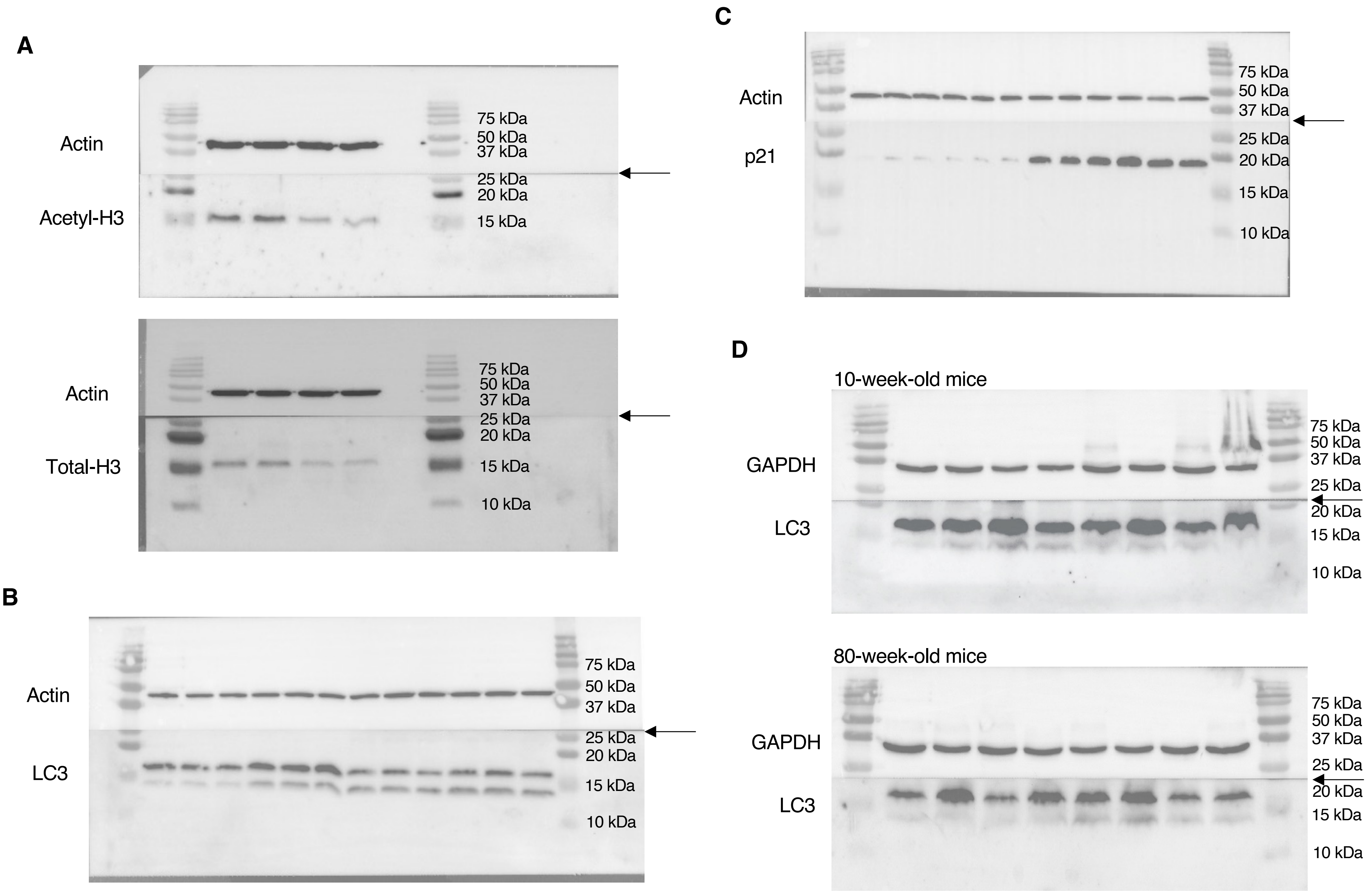

Supplementary Figure-7.
Uncropped blots for Fig. 2A (A), Fig. 2B (B), Supplementary Fig. 1A (C), and Supplementary Fig. 6A (D). Membranes were cut into 2 pieces at the position shown by arrows before incubation with each 1st antibody.
